# A Literature Review on Oral Lumenoscopy: A Non-invasive Method for Diagnosing Oral Potentially Malignant Disorders

**DOI:** 10.7759/cureus.75788

**Published:** 2024-12-16

**Authors:** Pavithra Dharumalingam, Divya Vinayachandran, Ganesh C, Shanthi M, Backiyalakshmi A, Haripriya Selvakumar

**Affiliations:** 1 Oral Medicine and Radiology, SRM Kattankulathur Dental College and Hospital, SRM Institute of Science and Technology (SRMIST), Chennai, IND

**Keywords:** chemi-illumination, oral cancer, oral lumenoscopy, premalignant lesions, vizilite

## Abstract

Dentistry still faces difficulties in diagnosing oral precancer and cancer, especially when it comes to early phase changes or disease detection, evaluation, and treatment. In essence, oral lumenography is the process of identifying oral lesions using a chemiluminescent light source and a toluidine blue labeling system. Since neoplastic epithelial cells have a changed nuclear-cytoplasmic ratio, acetic acid dehydration brings out this nuclear density and gives the tissue an "acetowhite" look. This phenomenon is further intensified when diffuse blue-white chemiluminescent illumination is created by using toluidine blue, which preferentially stains premalignant lesions, in place of ordinary lighting. In developing countries like India, oral cancer and other precancerous and malignant lesions of the oral cavity are very common. The most common diagnostic methods for oral mucosal lesions suggestive of malignancy or premalignancy are tissue samples and histological examination. There is a waiting period following this invasive operation before learning the diagnostic findings. Thus, the creation of a non-invasive screening instrument is required to detect oral cancer. Thus, the main goal of our work is to create a real-time, non-invasive diagnostic tool for oral cancer screening that is based on optical imaging methods like fluorescence emission imaging and diffuse reflectance imaging. To enhance clinical assessment and facilitate the diagnosis of premalignant and early-stage malignant lesions, numerous novel approaches have been developed. In this article, the purpose of a tissue reflectance-based analysis has been discussed. It is currently offered for sale under the ViziLite brand (Zila Pharmaceuticals, Phoenix, AZ, US) after being altered for usage in the oral cavity.

## Introduction and background

Oral cancer is the 10th most common type of cancer worldwide and is one of the most debilitating and disfiguring diseases. Oral squamous cell carcinoma (OSCC) accounts for 90% of the oral malignancies present in the world [1,2]. In wealthy countries, cancer is the second most common cause of death. According to age-standardized incidence, there are 12.6 cases of oral cancer for every 100,000 individuals in India [3].

The five-year relative survival rate for OSCCs in stages I and II is over 80%, while it is less than 30% for stages III and IV, which represent advanced stages of the illness [4]. Furthermore, more aggressive therapy is needed for advanced disease, involving a combination of medicines that may increase morbidity, increase healthcare costs, and lower quality of life. Increasing the diagnosis of suspicious oral premalignant lesions (OPLs) and early OSCC detection is the most sensible strategy to reduce morbidity and death related to OSCCs. The international literature has used the phrases "precancer," "precursor lesions," "premalignant," "intra-epithelial neoplasia," and "potentially malignant" to refer to a broad range of clinical appearances that may develop into cancer.

According to estimates from the American Cancer Society, 42% of leukoplakias with dysplasia will eventually undergo malignant degeneration, and up to 25% of leukoplakias are premalignant or malignant. By monitoring patients with OPLs for a maximum of 18.6 years (average 6.8 years), Holmstrup et al. discovered that non-homogeneous leukoplakia posed a sevenfold higher risk of malignant degeneration in comparison to homogenous leukoplakia. A lesion's chance of becoming malignant increased by 5.4 times when its size exceeded 2 cm. At the time of the first biopsy, 70%-90% of erythroplakia cases were found to be clearly malignant or significantly dysplastic [5].

It has been suggested that oral lumenoscopy, which is based on the tissue reflectance property, can improve the oral screening techniques now in use by helping to detect, assess, and track abnormalities of the oral mucosa. These are purportedly useful in identifying early carcinogenic mucosal alterations that may be invisible to the naked eye in order to evaluate the biological potential of clinically aberrant mucosal lesions [6].

## Review

Chemiluminescence

Early observations of luminescence in living things, or bioluminescence, date back to 1500 BC in Chinese literature. The most known examples of this natural occurrence include glowworms and fireflies. A German physician named Henning Brand first documented artificial light (chemiluminescence) in 1669 when he reported his discovery of phosphorus. Eilhardt Weidemann first introduced the word "chemiluminescence" in 1888 [7].

Luminescence is the release of light from an excited state of a molecule as it relaxes to its ground state. The energy source used by the different types of luminescence to attain the excited state varies. Electromagnetic radiation (photoluminescence, sometimes called fluorescence or phosphorescence) can provide this energy, as can heat (pyroluminescence), the impact of electrons (cathodoluminescence), frictional forces (triboluminescence), or crystallization (crystalloluminescence). The energy used in chemiluminescence is produced by a chemical reaction [7].

When the exogenic reaction's vibronically stimulated product relaxes to its ground state and emits photons, this phenomenon is seen. An electron can change from its ground state to an excited electronic state when the chemical reaction generates sufficient energy. Different hues and intensities of light throughout the visible spectrum are produced by chemiluminescent processes. Although there are additional chemiluminescence systems, the peroxy-oxalate and luminol-based systems are the most often used [7].

Chemiluminescence has long been utilized by the obstetrics and gynecology department as an extra tool for the early detection of precancer and cervical cancer. This technique, known as "speculoscopy," uses chemiluminescent light based on the peroxyoxalate system to inspect the cervix following the application of 5% acetic acid.

In order to detect oral precancer and cancer, a new technique called lumenoscopy, or ViziLite (Zila Pharmaceuticals, Phoenix, AZ, US), was recently introduced to the field of oral oncology. The US Food and Drug Administration (FDA) authorized ViziLite® as an adjunctive technology in November 2002 [8].

In June 2004, the American Dental Association's Code Revision Committee assigned ViziLite a dental reimbursement code. The FDA approved the ViziLite Plus system, formerly known as the ViziLite blue oral lesion identification and marking system, in November 2004 [9]. ViziLite Plus is the result of combining ViziLite utilizing the oral lesion marking system toluidine blue. Merging the blue toluidine marking system with chemiluminescent technology increases the visibility of oral lesions. An FDA 510(k) approval may be obtained by a medical device manufacturer whose product is "substantially equivalent" to one that was sold before 1976.

For oral chemiluminescence, the following brands are available: ViziLite, MicroLux DL (AdDent Inc., Danbury, CT, US), and ViziLite Plus. A single-use, disposable chemiluminescent light package powers the MicroLux device, but the ViziLite Plus uses a rechargeable battery-powered light source. Chemiluminescence as a screening technique for oral cancer has been thoroughly investigated. Despite the fact that just one study utilizing MicroLux DL has been published, given how similar the two technologies' emission characteristics are, it is unlikely that the study's conclusions will differ significantly [10].

Components

The ViziLite kit is a one-time-use item that includes a retractor (sheath and handle), a light-emitting capsule, and a 1% acetic acid solution. The ViziLite capsule, also known as the chemiluminescent light stick, is made out of an inner glass vial that contains hydrogen peroxide and an exterior shell made of a flexible plastic cap that comprises aspirin or acetylsalicylic acid. The capsule releases hydrogen peroxide when it is flexed, causing the fragile glass vial within to burst. Light with a wavelength of between 430 and 580 nm is produced by the reaction of chemicals in the outer and inner compartments. The duration of the lighting is roughly 10 minutes. Purified water, acetic acid, sodium benzoate, raspberry flavor, propylene glycol base, and alcohol make up the ViziLite 1% acetic acid solution. Three swab pieces make up ViziLite Plus: two swabs with a 1% acetic acid rinse, one with a toluidine blue metachromatic vital tissue dye solution, and one with a post-dye decolorizer. The deep blue hue toluidine blue produces makes the chemiluminescent positive area easier to notice and identify [6].

ViziLite

An option for white light illumination for visual inspection is the ViziLite system. It highlights oral tissue in blue light using a disposable chemiluminescent light source. It uses a single-use, disposable chemiluminescent light stick with wavelengths of 430, 540, and 580 nm. A 30-second acetic acid prerinse is required to dry the tissue before utilizing the ViziLite Plus with a toluidine blue system. The goal of using this light stick is to make it easier to see the differences between oral white lesions and normal mucosa. Normal epithelium absorbs light and appears dark, whereas dysplastic or hyperkeratinized lesions appear white. The nuclear content and mitochondrial matrix are more densely packed in the sick tissues, which preferentially reflect light. These variables might be connected to the variation in color or the changed thickness of the epithelium.

The principle utilized in ViziLite has several limitations, including low specificity, which often results in false positives and limits its ability to accurately distinguish normal from abnormal tissues. It is unable to differentiate between inflammatory, benign, and malignant lesions, making it challenging to assess the severity or nature of abnormalities. Furthermore, its diagnostic value is questionable due to the lack of reliable histopathological correlation, highlighting the need for adjunctive diagnostic methods such as biopsy or advanced imaging to confirm findings and ensure accuracy.

How does it work?

Oral lumenoscopy works on the property of tissue reflectance. Nuclear-cytoplasmic ratios are frequently changed in neoplastic epithelial cells. This nuclear density is highlighted by acetic acid dehydration, which also gives tissue an acetowhite color. By substituting diffuse blue-white chemiluminescent lighting for conventional lighting, this phenomenon can be increased even more. In contrast, abnormal cells have an increased nucleus:cytoplasm ratio, an epithelium with excessive keratinization, hyperparakeratinization, and/or significant inflammatory infiltration, and they also absorb light and appear bluish. These cells also seem acetowhite and have borders that are brighter and more defined.

One component of lumenoscopy is a chemiluminescent light source. There is a peroxyoxalate solution inside the light stick. The capsule is made up of an inside glass vial and an exterior shell made of flexible plastic. The acetylsalicylic acid in the outer capsule and the hydrogen peroxide in the inner vial react to produce a bluish-white light that lasts for around 10 minutes and has a wavelength of 430-580 nm. To further assess and closely monitor changes in lumenoscopy-identified lesions that are visible in normal light, toluidine blue is utilized. The deep blue staining is provided by toluidine blue, which comes in an easy-to-use three-swab container [6].

Mechanism of action

Oral lumenoscopy, such as ViziLite, enhances the detection of dysplastic and neoplastic oral lesions by identifying changes in mucosal tissue composition through light absorption and reflection. Acetic acid solution dehydrates the cytoplasm, clearing debris and allowing light to pass through the epithelium. This causes abnormal cells to reflect light and appear as "acetowhite," a visual marker of potential malignancy. The refractive properties of cells are altered, making dysplastic and neoplastic lesions more visible under chemiluminescent light, aiding in early detection [11].

The ViziLite system utilizes acetylsalicylic acid and hydrogen peroxide to excite a fluorescent dye, which emits light when electrons return to a lower energy state, helping visualize abnormal tissue. Additionally, toluidine blue dye is used to stain dysplastic cells, further highlighting high-risk areas. Combined with conventional oral mucosal exams, chemiluminescence improves the identification and monitoring of potentially cancerous lesions in the mouth. This technology also has broader applications, including pharmaceutical analysis, gene assays, and chemiluminescence imaging, making it a versatile tool for both diagnostic and research purposes [12].

Procedure

Step 1

Encourage the patient to read the patient leaflet in the reception area. Ask the patient to sign a consent form and place it in the patient’s file.

Step 2

Examine the oral cavity routinely, noting any lesions that may be present. Use the 1% acetic acid lumenoscopy prerinse solution on the patient for 30-60 seconds. The 1% acetic acid wash is intended to eliminate surface debris and, perhaps as a result of minor cellular dehydration, will make epithelial cell nuclei more visible.

Step 3

Bend the flexible outer light stick to get the brittle inner wall to break and shake vigorously to mix the contents. Insert the light stick into the open end of the retractor and assemble. Dim the room lights or use the provided eyewear to facilitate the examination.

Step 4

Re-examine the oral cavity using a lumenoscopy device. The open retractor window should face the tissue being examined. Look for any abnormalities and document them on the mouth map located on the back of the patient consent form.

Step 5

Apply the toluidine blue marking system to the lesion visible under lumenoscopy illumination. Toluidine blue is made up of three separate swabs: one with the metachromatic vital tissue dye toluidine blue and two with a 1% acetic acid rinse and a post-dye decolorizer. Swabs should be applied in a sequential order labeled 1, 2, and 3.

Step 6

Lesions stained with toluidine blue can be viewed clearly even without a lumenoscopy device.

Step 7

Now, take an intra-oral photograph for inclusion in the patient records, submission to the patient’s insurance company, or referral to a specialist.

The following advantages of this method are ease of use, safety, non-invasiveness, non-toxicity to biological tissues, and a reduced risk of cross-contamination due to its single use. In addition, it produces results instantly, learns quickly, and has minimal operator variability.

The major disadvantage of this method is that it can only be used once per patient, and it has a number of drawbacks, including cost, the requirement for a dark environment, the inability to identify the best biopsy site, the inability to measure the visualization results objectively, and an unknown level of sensitivity and specificity [13,14].

Emerging technologies

Brush Biopsy Technique

Biopsies are tissue samples taken from living bodies in order to determine the nature of a tumor and whether it is benign or malignant. By using a brush to capture epithelial cells and evenly apply the samples to a glass slide, the brush biopsy approach allows for computerized microscopic examination and imaging system follow-up. The term “oralcdx” refers to this entire system. Trans-epithelial cell sample collection from mucosal lesions is part of the oralcdx brush system approach. Dental professionals can use this to detect mouth cancer in its early stages. When used on low-risk patients, a brush biopsy leads to more false-positive results and is less accurate in identifying dysplastic alterations in the high-risk population [15].

VELscope System

It is produced by LED Medical Diagnostic Inc. Light-emitting diodes (LEDs) with a wavelength of 400-460 nm are used in this portable fluorescence imaging equipment to display tissue fluorescence. The normal mucosa in the oral cavity emits a pale green autofluorescence when exposed to blue light, but abnormal tissues show reduced autofluorescence and appear darker than healthy tissue. In a study led by Poh and colleagues, 97% sensitivity and 94% specificity were achieved in identifying premalignant lesions [15,16].

Identafi

It is a transportable, tiny gadget in the shape of a dental mirror that is produced by Trimira™ LLC (Marietta, GA, US). The tool improves imaging of mucosal abnormalities by utilizing reflectance and fluorescence approaches. To evaluate the reflectance of the tissue, three different light wavelengths are produced using Identafi technology using LEDs: amber, violet, and white. The goal of the amber light is to increase the oral mucosa's ability to reflect light, which will facilitate the differentiation of normal from aberrant tissue vasculature. In research by Schwarz et al., the Identafi 3000 was used to screen 124 patients. The results showed 82% sensitivity and 87% specificity in separating oral disorders that were cancerous from non-cancerous ones [15].

Oscan

A smartphone mouth positioner mounting made of plastic was developed by the Prakash Lab at Standford University to enable early oral cavity diagnosis. It consists of a plastic mouth positioner with two rows of brilliant blue LED lights and a circuit board that connects to any smartphone. This screening method makes use of the smartphone's built-in camera, which enables the user to capture a panoramic, high-resolution image of the oral cavity. Doctors and dentists can receive images wirelessly for examination and diagnosis.

Optical Spectroscopy

This non-invasive technique measures the molecular and structural alterations of oral tissues as a result of neoplastic growth. Optical imaging and spectroscopic screening may enhance the diagnosis of oral cancer in addition to saving time and avoiding more intrusive treatments. Two different modalities are used in optical imaging systems: diffuse reflectance techniques and fluorescence emission techniques. These techniques provide tissue morphology data that aids in the identification of malignancy. Each of these spectroscopic methods has a basis in physics and can potentially support more well-established methods for cancer detection [15].

Fluorescence Technique

It is an innovative method to distinguish between healthy and malignant cells. UV and visible light are absorbed by fluorophores found in the oral epithelium and stroma. These cells then partially re-emit the light at a longer wavelength as fluorescence. It is feasible to see the long wavelength fluorescence of reflected light with the unaided eye. These modifications affect tissue fluorophores' distribution and the way they glow in response to 400-460-nm blue excitation light stimulation. This method has been extensively researched and has numerous benefits, including non-invasiveness, real-time diagnosis, and increased efficacy of oral examinations. A scatterplot was utilized to differentiate between normal oral mucosa and hyperplasia, dysplasia, and squamous cell carcinoma (SCC) using these fluorescence intensity ratios. For the F500/F685 intensity ratio, sensitivity and specificity were 100, whereas for the F500/F635 intensity ratio, they were 1,005 and 83%, respectively [17]. 

Diffuse Reflectance Technique

It is a non-invasive, cost-effective, and straightforward method for diagnosing tissue abnormalities among the emerging quantitative optical techniques. After entering the tissue, white light undergoes a sequence of elastic scattering and observation processes before being discharged once more as diffusely reflected light. In order to identify the dysplastic transformation of the tissue, it is studied to find the spectral sign of the tissue. With reflectance spectroscopy, cancer is detected by changes in tissue's reflectance spectrum caused by dysplastic tissue and other optical features including coefficients of absorption and scattering. These substances all have various wavelength-dependent absorption coefficients. The optical window for diagnostic applications is defined as the wavelength range between 600 and 1,300 nm. This region allows light to enter more easily.

Some lesions of diagnostic importance

Oral Squamous Cell Carcinoma

OSCC epidemiology: More than 90% of oral cancers are OSCCs, which originate in the mucosal lining of the oral cavity. Worldwide, there are about 500,000 new cases diagnosed annually. Risk factors are connected to the growth of SCC in the mouth. OSCC has multiple underlying causes. It has not been possible to identify or accept a single causative agent or component. The malignant metamorphosis is most likely the result of several causes working together. It is commonly known that there is a strong link between tobacco use, alcohol intake, and OSCC [18].

Oral lumenoscopy, such as ViziLite, plays a valuable adjunctive role in the investigation of OSCC by enhancing the visualization of dysplastic or potentially malignant changes. It highlights suspicious areas, including high-risk lesions like erythroleukoplakia, which are more likely to progress to OSCC. This tool is particularly useful for detecting subtle or flat lesions not visible under standard lighting and for delineating lesion margins, aiding in targeted biopsy and surgical planning. Although oral lumenoscopy improves the identification of suspicious areas, it cannot distinguish between inflammatory, benign, or malignant lesions, making histopathological evaluation essential for definitive OSCC diagnosis and treatment planning [18].

Oncogenic viruses: These viruses are able to alter their genetic makeup, which hinders the host's ability to control the proper division and growth of the infected cell. According to a recent study, there may be a tiny correlation between human papillomavirus (HPV) and several oral and oropharyngeal malignancies. It has been discovered that HPV-16 or HPV-18 is present in up to 50% of OSCCs that begin in Waldeyer's tonsillar ring and 15%-20% of those that form in the tongue and other oral cavity regions.

Precancerous lesions

Premalignant lesions of the oral cavity invasive OSCC typically manifest prior to clinically significant alterations in the oral mucosa that signify precancerous conditions. Premalignant epithelium alterations are often seen in OSCC patients; epithelial dysplasia is considered to be the most significant of these changes [19].

Carcinogenesis is most likely to arise from severe dysplasia. Precancerous lesions can vary in severity from mild to severe based on their histopathological findings. It is difficult to anticipate which precancerous tumors may progress to oral carcinoma. Cancer develops from dysplastic epithelial lesions in about 16% of cases, and the disease can progress from months to more than 20 years. Dysplasia is not always a sign of the development of SCC and can occur even in the absence of it [19].

Leukoplakia

Leukoplakia is a clinical word that refers to "a white patch or plaque that cannot be characterized clinically or pathologically as any other disease," according to the World Health Organization. The mucosa's natural response to long-term irritation or damage accounts for the bulk of leukoplakic lesions. Unfitting dentures and unhealthy dental habits including biting the tongue or cheeks are common causes. These white mucosal lesions can be caused by a variety of factors, including syphilis, chronic hyperplastic candidiasis, mechanical and chemical irritants, and electrogalvanic responses [20].

The most common locations for leukoplakic lesions are the floor of the mouth, the lateral and ventral borders of the tongue, the labial and buccal mucosa, the gingiva, the soft palate, and the retromolar regions. Eighty percent of leukoplakias are benign. This is the great majority. The residual lesions are classified as either malignant or premalignant (carcinoma in situ or dysplastic) [20].

ViziLite is an effective tool for diagnosing leukoplakia by enhancing the visualization of dysplastic changes that may not be visible under standard lighting. It is particularly useful for detecting subtle or flat leukoplakic lesions and identifying high-risk areas that have a higher potential for malignant transformation into OSCC. The technique also helps define the margins of lesions, facilitating precise biopsy or surgical planning.

Erythroleukoplakia

Similar correlations have been shown between a higher risk of oral cancer and leukoplakia and erythroplakia. The terms "speckled leukoplakia" and "speckled erythroplakia" are only two of the several designations for these reddish-white hybrid lesions. The probability of these lesions developing into malignant changes is four times higher than that of homogeneous leukoplakias. Erythroleukoplakia can occur anywhere within the oral cavity. It is more skewed toward men. It should come as no surprise that these lesions are commonly found in patients who smoke, drink, or practice poor dental hygiene [21].

Candida albicans

These lesions often harbor candidal infections that may contribute to dysplastic alterations. However, studies have not established a definitive connection between candidal involvement and malignant transformation. Mixed red and white lesions, particularly those persisting for 7-14 days after excluding localized causes, should be carefully evaluated and considered for biopsy due to their higher likelihood of progressing to cancer [22].

Erythroplakia

The clinical word "erythroplakia" is used to describe a velvety red area that defies easy categorization. These lesions are first identified during a normal dental examination and are frequently asymptomatic. It is required to do a biopsy on erythroplakic lesions since, according to histology, 90% of these lesions indicate either cancer in situ, severe dysplasia, or malignancy. The patient needs to be closely monitored because there is a chance that multiple oral cavity sites can be impacted-a situation called "field cancerization" [6].

Oral Submucous Fibrosis

Oral submucous fibrosis (OSF) is characterized by fibrosis of the mucosa lining the upper digestive tract. Atrophic idiopathic mucosa oris is the name Schwartz gave to the oral fibrosing condition he found in five Indian women from Kenya in 1952. In 1953, Joshi later came up with the term OSF to describe the illness.

The typical clinical picture, except for the early stages of the disease, is brought on by lamina propria and submucosa fibrosis and a progressive decrease in tissue mobility. The primary ingredient in betel quid, areca nut chewing, is especially linked to OSF. Chewing areca nuts, eating chilies, immunological and genetic processes, consuming inadequate nutrition, and other factors have all been linked to the development of OSF [23].

Discussion

ViziLite technology is being used to diagnose precancerous and malignant tumors of the oral cavity. Chemiluminescent light can be used to diagnose dysplasia because of certain characteristics. Low-level multichromatic light highlights the reflecting difference between healthy and sick epithelium. Tissues with differing densities (nuclear-cytoplasmic ratios) absorb or reflect it differently. The photo-enhanced chemiluminescence effect, low-level multichromatic light, and low-level light output with minimal glare are a few examples of these. Three spectrum emission peaks between 430 and 580 nm are visible in the light, which has a peak intensity of less than 60 lumens. The examiner sees it as blue-white due to its multichromatic and low intensity. Any mucosal surface that is surrounded by cylindrical chemiluminescent capsules experiences uniform, shadow-free sheets of light.

The effectiveness of ViziLite in oral cancer prediagnosis and detection has only been studied a limited amount. The results, conclusions, and outcomes of these investigations have varied. Lesions that were missed by a routine eye examination could be found with great efficiency with ViziLite.

It has been discovered that ViziLite is a more accurate tool for identifying leukoplakia than red lesions and erythroplakia. This method improved and made leukoplakia more visible. In one study, this result was significant (p < 0.01). Only half of the erythroplakia exhibited acetowhitening, compared to over 80.3% of leukoplakia. Both red and white lesions exhibited increased sharpness and brightness.

Leukoedema, frictional keratosis, fibroma, fibroepithelial hyperplasia, lichen planus, ulcerative lesions linked to trauma, linea alba, and leukoedema were among the other positive lesions. Additionally positive was epithelium with hyperkeratinization, hyperparakeratinization, or chronic inflammatory infiltration. In such circumstances, the test's specificity was significantly decreased.

Few individuals believe that it would be useful for screening and monitoring cases of previously treated oral cancer. As the majority of the writers have indicated, more research linking ViziLite to changes in the cytology, histology, clinical state, and molecular makeup is crucial to assessing the sensitivity and specificity of this method. For it to be more effective, the technological parts need to be improved (Table 1).

**Table 1:** Studies on ViziLite on oral cancer detection

S. No.	Authors	Topic	Focus	Result
1	Huber et al.	Acetic acid wash and chemiluminescent illumination as an adjunct to conventional oral soft tissue examination for the detection of dysplasia: a pilot study	Pilot study on oral tissues under chemiluminescent light	Blue-white chemiluminescent light is significantly reflected by epithelium that has undergone hyperkeratinization, an altered nuclear-to-cytoplasmic ratio, or chronic inflammation. This causes lesions to look noticeably magnified and "acetowhite" [24].
2	Mehrotra et al.	A cross-sectional study evaluating chemiluminescence and autofluorescence in the detection of clinically innocuous precancerous and cancerous oral lesions	Comparison of ViziLite and VELscope	The ViziLite was used to assess and biopsy 102 lesions. None of the 3 dysplasias and 1 cancer that they discovered were picked up by the ViziLite. A VELscope was used to evaluate 156 biopsied lesions. Six of the 11 dysplasias and one cancer were identified with VELscope [25].
3	Ram and Siar	Chemiluminescence as a diagnostic aid in the detection of oral cancer and potentially malignant epithelial lesions	Comparison of ViziLite and toluidine blue	ViziLite was superior in identifying asymptomatic and non-evident lesions for biopsy locations. It has better sensitivity and accuracy compared to tolonium chloride [26].
4	Epstein et al.	Analysis of oral lesion biopsies identified and evaluated by visual examination, chemiluminescence and toluidine blue	ViziLite and toluidine blue	Chemiluminescence helps discover mucosal lesions that are missed by conventional inspection by increasing brightness and margin visibility. By specifically identifying cancer and dysplastic tissues while preserving a 100% negative predictive value, toluidine blue staining substantially lowers false-positive biopsies. These methods increase the precision of diagnosis for possibly cancerous oral lesions [27].
5	Sharma and Khan	Non-invasive diagnostic tools in early detection of oral epithelial dysplasia	Comparison of exfoliative cytology, toluidine blue, and ViziLite	When compared to toluidine blue, exfoliative cytology had moderate sensitivity (50%) and high specificity (83.3%), but when exposed to chemiluminescent light, it demonstrated a reduced sensitivity (42.9%) and specificity (79.3%). Compared to toluidine blue, chemiluminescent light showed greater sensitivity (60%) but poorer specificity (70%), underscoring the complementing functions of these instruments in identifying dysplasia in leukoplakia [28].

The unwanted processes that result from the opposing chemical reaction must be overcome. These processes include internal conversion, breakdown and intermediate formation, collisional deactivation, inter-system crossing, and the loss of the excited molecule due to chemical interactions [29].

Oral lumenoscopy has remarkable potential when combined with advanced technologies like AI, the metaverse, augmented reality (AR), and virtual reality (VR). AI algorithms can enhance diagnostic accuracy by analyzing lumenoscopic images to detect minimal changes and predict the progression of conditions, aiding the physician in determining the treatment plan [30]. The metaverse provides virtual consultation spaces and interactive education, helping patients understand their diagnoses and engage in their care [31]. AR supports clinicians in real time by overlaying diagnostic data onto live views, allowing for more precise examinations. VR provides immersive training simulations for practitioners to refine diagnostic skills on virtual patients and collaborate on complex cases. Blockchain application further ensures secure handling of patient data, fostering trust in digital diagnostics [32]. Altogether, these technologies combined with oral lumenoscopy open a new pathway for early detection, patient empowerment, and improved outcomes in managing premalignant oral disorders [33].

## Conclusions

It appears that making it easier to identify lesions that may be malignant is the most effective way to reduce the morbidity and death rate related to mouth cancer. It is widely acknowledged that mouth cancer may not develop from a premalignant lesion if it is identified early and treated appropriately. Clinical tools can help practitioners see suspected oral lesions that are difficult to see during a traditional oral examination.

Oral cancer diagnostic tools have a bright future ahead of them, with the goal of improving the standard of patient care delivered by every physician. With advancements in technology and methods, an imaging modality could eventually be able to eliminate the need for a sample, define the surgical margins, and give a clear assessment of how well oral cancer is being treated. While ViziLite has demonstrated efficacy in the early identification of oral cancer, this benefit is not great enough to outweigh the expense of the test. Oral lumenoscopy increases the diagnostic yield and broadens mouth screening beyond previous levels by highlighting precancerous and cancerous lesions.

## References

[1] Badwelan M, Muaddi H, Ahmed A, Lee KT, Tran SD (2023). Oral squamous cell carcinoma and concomitant primary tumors, what do we know? A review of the literature. Curr Oncol.

[2] Chi AC, Day TA, Neville BW (2015). Oral cavity and oropharyngeal squamous cell carcinoma--an update. CA Cancer J Clin.

[3] Verma R, Singh A, Chowdhury N, Joshi PP, Durgapal P, Rao S, Kishore S (2022). Evaluation of histomorphological parameters to predict occult nodal metastasis in early-stage oral squamous cell carcinoma. Turk Patoloji Derg.

[4] Jäwert F, Nyman J, Olsson E, Adok C, Helmersson M, Öhman J (2021). Regular clinical follow-up of oral potentially malignant disorders results in improved survival for patients who develop oral cancer. Oral Oncol.

[5] Holmstrup P, Vedtofte P, Reibel J, Stoltze K (2006). Long-term treatment outcome of oral premalignant lesions. Oral Oncol.

[6] Srivastava R, Devi MP, Garg K, Jyoti B (2012). Oral lumenoscopy-visualizes what your eyes can't. Journal of Oral Sign.

[7] Araujo-Filho JL, Melo-Junior MR, Carvalho LB Jr (2011). Potential applications of the chemiluminescent methods in tumoral diseases investigation. Int J Pharma Biosci.

[8] Trullenque-Eriksson A, Muñoz-Corcuera M, Campo-Trapero J, Cano-Sánchez J, Bascones-Martínez A (2009). Analysis of new diagnostic methods in suspicious lesions of the oral mucosa. Med Oral Patol Oral Cir Bucal.

[9] Oh ES, Laskin DM (2007). Efficacy of the ViziLite system in the identification of oral lesions. J Oral Maxillofac Surg.

[10] Sambandham T, Masthan KM, Kumar MS, Jha A (2013). The application of ViziLite in oral cancer. J Clin Diagn Res.

[11] Borkar S, Reche A, Paul P, Deshpande A, Deshpande M (2023). Noninvasive technique for the screening and diagnosis of oral squamous cell carcinoma. Cureus.

[12] Vashisht N, Ravikiran A, Samatha Y, Rao PC, Naik R, Vashisht D (2014). Chemiluminescence and toluidine blue as diagnostic tools for detecting early stages of oral cancer: an invivo study. J Clin Diagn Res.

[13] Shashidara R, Sreeshyla HS, Sudheendra US (2014). Chemiluminescence: a diagnostic adjunct in oral precancer and cancer: a review. J Cancer Res Ther.

[14] Aggarwal A, Ammanagi R, Keluskar V (2011). Oral lumenoscopy: an adjuvant in early screening of oral cancer. J Indian Acad Oral Med Radiol.

[15] Gupta P (2017). An update on light-based technologies and fluorescent imaging in oral cancer detection. Oncobiol Targets.

[16] Abdul NS (2023). Role of advanced diagnostic aids in the detection of potentially malignant disorders and oral cancer at an early stage. Cureus.

[17] Mallia RJ, Thomas SS, Mathews A, Kumar R, Sebastian P, Madhavan J, Subhash N (2008). Laser-induced autofluorescence spectral ratio reference standard for early discrimination of oral cancer. Cancer.

[18] Priyanka AD, Narayanan S, Mata B (2016). A survey paper on development of screening device for oral cancer detection. Int J Sci Tech Res Eng.

[19] Cheng YS, Wright J (2011). Advances in diagnostic adjuncts for oral squamous cell carcinoma. Open Pathol J.

[20] Mascitti M, Orsini G, Tosco V (2018). An overview on current non-invasive diagnostic devices in oral oncology. Front Physiol.

[21] Awan KH, Morgan PR, Warnakulasuriya S (2011). Utility of chemiluminescence (ViziLite™) in the detection of oral potentially malignant disorders and benign keratoses. J Oral Pathol Med.

[22] Shin D, Vigneswaran N, Gillenwater A, Richards-Kortum R (2010). Advances in fluorescence imaging techniques to detect oral cancer and its precursors. Future Oncol.

[23] Mojsa I, Kaczmarzyk T, Zaleska M, Stypulkowska J, Zapala-Pospiech A, Sadecki D (2012). Value of the ViziLite Plus System as a diagnostic aid in the early detection of oral cancer/premalignant epithelial lesions. J Craniofac Surg.

[24] Huber MA, Bsoul SA, Terezhalmy GT (2004). Acetic acid wash and chemiluminescent illumination as an adjunct to conventional oral soft tissue examination for the detection of dysplasia: a pilot study. Quintessence Int.

[25] Mehrotra R, Singh M, Thomas S, Nair P, Pandya S, Nigam NS, Shukla P (2010). A cross-sectional study evaluating chemiluminescence and autofluorescence in the detection of clinically innocuous precancerous and cancerous oral lesions. J Am Dent Assoc.

[26] Ram S, Siar CH (2005). Chemiluminescence as a diagnostic aid in the detection of oral cancer and potentially malignant epithelial lesions. Int J Oral Maxillofac Surg.

[27] Epstein JB, Silverman S Jr, Epstein JD, Lonky SA, Bride MA (2008). Analysis of oral lesion biopsies identified and evaluated by visual examination, chemiluminescence and toluidine blue. Oral Oncol.

[28] Sharma N, Khan M (2011). Non-invasive diagnostic tools in early detection of oral epithelial dysplasia. J Clin Exp Dent.

[29] Nagi R, Reddy-Kantharaj YB, Rakesh N, Janardhan-Reddy S, Sahu S (2016). Efficacy of light based detection systems for early detection of oral cancer and oral potentially malignant disorders: systematic review. Med Oral Patol Oral Cir Bucal.

[30] Sawhney H, Bhargava D, Kashwani R, Mishra R (2023). Artificial intelligence as a tool for improving oral cancer outcomes. Arch Dent Res.

[31] Kashwani R, Jose AT, Gambhir S, Virk S, Roy S (2024). The role of the metaverse in revolutionizing dental practice: implications across all departments. Int Dent J Stud Res.

[32] Kashwani R, Sawhney H (2023). Dentistry and metaverse: a deep dive into potential of blockchain, NFTs, and crypto in healthcare. Int Dent J Stud Res.

[33] Kashwani R, Ahuja G, Narula V (2024). Future of dental care: integrating AI, metaverse, AR/VR, teledentistry, CAD & 3D printing, blockchain and CRISPR innovations. Community Pract.

